# Role of Quinone Reductase 2 in the Antimalarial Properties of Indolone-Type Derivatives

**DOI:** 10.3390/molecules22020210

**Published:** 2017-01-30

**Authors:** Laure-Estelle Cassagnes, Nambinina Rakotoarivelo, Serena Sirigu, Pierre Pério, Ennaji Najahi, Léonard M. G. Chavas, Andrew Thompson, Régis Gayon, Gilles Ferry, Jean A. Boutin, Alexis Valentin, Karine Reybier, Françoise Nepveu

**Affiliations:** 1 UMR 152 Pharma-Dev, Université de Toulouse, IRD, UPS, 31062 Toulouse, France; laureestelle.cassagnes@gmail.com (L.-E.C.); nambine0508@yahoo.fr (N.R.); pierre.perio@univ-tlse3.fr (P.P.); najahimco@yahoo.fr (E.N.); alexis.valentin@univ-tlse3.fr (A.V.); karine.reybier-vuattoux@univ-tlse3.fr (K.R.); francoise.nepveu@univ-tlse3.fr (F.N.); 2 Synchrotron SOLEIL, L’Orme des Merisiers, BP 48 Saint-Aubin, 91190 Gif sur Yvette CEDEX, France; serena.sirigu@synchrotron-soleil.fr (S.S.); leonard.chavas@synchrotron-soleil.fr (L.M.G.C.); andrew.thompson@synchrotron-soleil.fr (A.T.); 3 Vectalys S.A., Parc Technologique du Canal, Bâtiment Canal Biotech 2, 3, Rue des Satellites, 31400 Toulouse, France; regis.gayon@vectalys.com; 4 Pôle d’Expertise Biotechnologie, Chimie, Biologie, Institut de Recherches Servier, 125, Chemin de Ronde, 78290 Croissy sur Seine, France; gilles.ferry@servier.com

**Keywords:** malaria, inhibitor, mechanism, human quinone reductase 2, indolones

## Abstract

Indolone-N-oxides have antiplasmodial properties against *Plasmodium falciparum* at the erythrocytic stage, with IC_50_ values in the nanomolar range. The mechanism of action of indolone derivatives involves the production of free radicals, which follows their bioreduction by an unknown mechanism. In this study, we hypothesized that human quinone reductase 2 (hQR2), known to act as a flavin redox switch upon binding to the broadly used antimalarial chloroquine, could be involved in the activity of the redox-active indolone derivatives. Therefore, we investigated the role of hQR2 in the reduction of indolone derivatives. We analyzed the interaction between hQR2 and several indolone-type derivatives by examining enzymatic kinetics, the substrate/protein complex structure with X-ray diffraction analysis, and the production of free radicals with electron paramagnetic resonance. The reduction of each compound in cells overexpressing hQR2 was compared to its reduction in naïve cells. This process could be inhibited by the specific hQR2 inhibitor, S29434. These results confirmed that the anti-malarial activity of indolone-type derivatives was linked to their ability to serve as hQR2 substrates and not as hQR2 inhibitors as reported for chloroquine, leading to the possibility that substrate of hQR2 could be considered as a new avenue for the design of new antimalarial compounds.

## 1. Introduction

The control and eradication of malaria require continuous efforts at several levels. The wide use of combined medicines associated with the use of insecticide-treated nets and indoor insecticide sprays have enabled significant progress in controlling malaria during the past decade [1]. It has been confirmed however that artemisinin efficacy has declined in South-East Asia, particularly in extensions of treatment for infectious diseases, old or new. These contemporary factors have altered the target profile for future antimalarial drugs to be discovered and developed in coming years [2,3].

Indolone-N*-*oxides (INODs) have antiplasmodial properties that target the asexual blood stage of *Plasmodium falciparum* (*P. falciparum*), with IC_50_ values (drug concentration required for 50% parasite growth inhibition in vitro = IC_50_) in the 1 to 100 nanomolar range [4]. Formulated as albumin-based nanoparticles, to overcome their low aqueous solubility in vivo, INODs strongly inhibited parasitaemia in a mouse model infected with *P. berghei* (99.1%) or in humanized mice parasitized with *P. falciparum* (99.6%) [5]. These compounds have several properties in healthy and parasitized erythrocytes, including bioreducibility [6], non-toxicity, and non-hemolytic properties [4]. Synthesis of a large variety of compounds with different oxidation states [7] (Figure 1), has demonstrated that only compounds that possessed the reducible N=C bond together with a pseudo-quinoid structure were active. This is the case of indolones (INDs) obtained by deoxygenation of the INODs. Further studies of the mechanisms of action of INODs on parasitized red blood cells (RBCs) showed that these molecules activated a Syk kinase cascade, which induced hyper-phosphorylation of band 3, a major protein in RBCs, and caused the host cell to burst [8]. To explain the activation of Syk kinase, it was proposed that these molecules had the ability to generate radical forms [9].

The cellular target responsible for INODs bioreduction and radical production is unknown, but reductase enzymes are strongly suspected. Graves and coworkers [10] have shown that the quinoline types of antimalarial drugs, such as chloroquine, primaquine, quinacrine, mefloquine, and quinine, acted by targeting quinone reductase 2 (hQR2) in human RBCs. Other studies showed that these molecules inhibited hQR2 in vitro [11]. It was also demonstrated that primaquine competitively inhibited the reducing co-factor, *N*-ribosidedihydronicotinamide NRH, and that chloroquine competed with the substrate (quinone) [12]. It was then hypothesized that an undertaking to discover or engineer potent hQR2 inhibitors might lead to new antimalarial drugs [13,14]. Hence, Choi et al. screened natural products with liquid chromatography in tandem with mass spectrometry (LC-MS) [15], and other teams designed and synthesized inhibitors of hQR2 [16,17,18,19]. However, assays conducted with hQR2 inhibitors showed antimalarial activity that was 50- to 100-fold lower than the activity obtained with chloroquine and its derivatives. These results suggested that no link existed between hQR2 inhibition and antiplasmodial activity during the erythrocyte stage of *P. falciparum.* Based on our previous studies, which demonstrated that hQR2 could reduce a large variety of quinone or pseudo-quinone compounds [20,21], we hypothesized that hQR2 could play a key role in the bioreduction of these indolone derivatives, because they were pseudo-quinone compounds. In this case, the indolone derivatives would act as substrates, not inhibitors, of hQR2. It is noteworthy that hQR2 has been specifically used to activate a pro-drug, called CB1954, which is an anti-cancer compound [22].

The present work aimed to shed light on the mechanism of action of indolone derivatives, and in particular, to determine the role of hQR2 in their antimalarial properties. We investigated interactions between hQR2 and representatives of the antimalarial series, INODs and INDs, and with other derivatives. The interaction was analyzed by studying the kinetic constants of the enzyme hQR2. We also determined the substrate/protein structure with X-ray diffraction analysis. The capacity of the enzyme to reduce the compounds was investigated by measuring the re-oxidation of the reduced form and the production of ROS, both on purified hQR2 or hQR2 overexpressed in cells.

## 2. Results and Discussion

The structures of all compounds tested are summarized in Figure 1.

### 2.1. Interactions of the Compounds with the hQR2 Enzyme

To evaluate the affinity of different compounds for hQR2, we determined the kinetic constants of a few representative compounds, including INODs (compounds **1** and **8**) and INDs (compounds **10** and **12**), as QR2 substrates. The water solubility of these compounds varied strongly from one molecule to another, ranging from 10 µg/mL (0.33 µM) for compound **1** to 1–3 mg/mL for compounds **8′**, **10**, and **12** (3 to 10 µM). Menadione, a natural substrate of hQR2, was used as the reference substrate to control the validity of the assay and to compare with the compounds tested. The *V*_max_ and *K*_M_ constants obtained with the different molecules are given in Table 1.

As expected, menadione was more rapidly catalyzed by hQR2 than the other tested compounds. Furthermore, the calculated *K*_M_ values for menadione were consistent with previous reports [23]. Note that QR2 has no activity in the absence of either substrate or co-substrate, strongly suggesting that it does not have any oxidase activity in the presence of O_2_ as electron acceptor. These results demonstrated that hQR2 had a high affinity for these molecules, but metabolized them at relatively slow rates.

**Figure 2:**
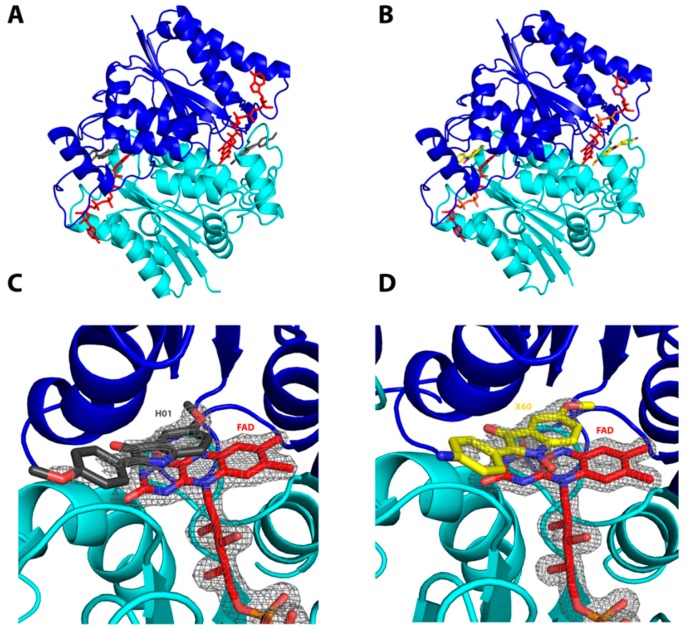
Structural illustrations of human quinone reductase 2 (hQR2) co-crystallized with compounds **8′** and **10**. (**A**,**B**) Crystal structure of the flavin adenine dinucleotide (FAD) complexed with hQR2, and bound to (**A**) compound **10** or (**B**) compound **8′** (cartoon representations were created with the Pymol program); (**C**,**D**) Detailed view of the ligands in the binding pocket (FAD, red sticks); (**C**) compound **10**, grey sticks; (**D**) compound **8′**, yellow sticks. Grey mesh represents the electronic density (2fo-fc map) surrounding FAD, compound **8′**, and compound **10** (1.5σ).

The most rapid transformations were obtained with the two most soluble compounds: compounds **8** and **12**. To confirm this interaction, we co-crystallized hQR2 with the chosen substrates, and subsequently determined the corresponding crystal structure. In this case, compounds **8′** and **10** were selected as representatives of the INOD and IND series, respectively, due to their high solubility in DMSO (solvent used to dissolve the compounds). Crystals of hQR2 in complex with flavin adenine dinucleotide (QR2-FAD) and bound to compound **8′** or to compound **10** adopted the orthorhombic lattice conformation (space group: *P*2_1_2_1_2_1_), with two molecules of hQR2-FAD and either compound **8′** (Figure 2B) or compound **10** (Figure 2A) per asymmetric unit.

The ligand residues could only be partially modeled in the electron density maps; in particular, no electron density was defined for the terminal anisolic ring, likely due to its flexibility and exposure to the solvent. The ligands (compounds **8′** and **10**) both occupied the same region of the protein; both were located above the FAD and interacted with the isoalloxazine ring through π–π stacking (Figure 2C,D). Compounds **8′** (Figure 2D) and **10** (Figure 2C) did not establish direct interactions with the protein residues, but interacted with water molecules present in the vicinity of the binding site (Table 2). From this series of data, there was no doubt that hQR2 could recognize these compounds as substrates. The structural data clearly showed that these compounds lingered in the catalytic site of the hQR2 protein, comparable to previously obtained data on co-crystallized substrates and inhibitors of hQR2 [24,25].

### 2.2. Free Radical Production during Metabolization of Indolone Derivatives by Purified hQR2

As previously demonstrated [20], the reduction of quinones by purified quinone reductase gave rise to the production of free radicals. This reaction evolved from the re-oxidation of hydroquinone and the concomitant electron transfer to oxygen, which produced superoxide radicals (Equation (1)).

(1) $$\begin{array}{ccc} Q & \overset{\mathrm{hQR2}}{\underset{\;}{\to }} & QH_{2},\\ QH_{2}+O_{2} & \to & {}^{●}QH+{O_{2}}^{-●}+H^{+}, \end{array}$$

**Figure 3:**
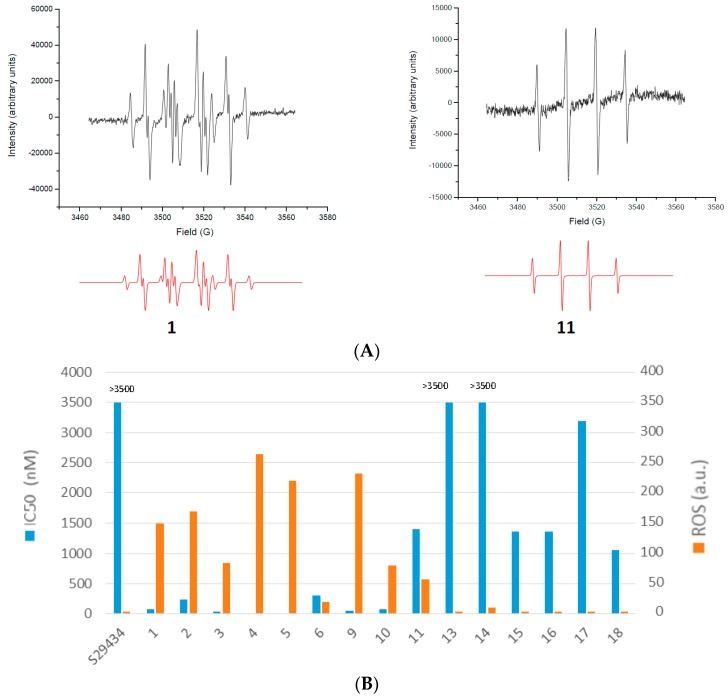
Free radicals produced by hQR2 metabolization of indolone derivatives. (**A**) Examples of EPR spectra recorded with the different compounds tested. Actual spectra (dark) were compared with theoretical spectra (red); (**B**) comparison of the compound potency (IC_50_ in nM, blue bars *P. falciparum*, strain FcB1) to the amount of radicals produced in a pure hQR2 system (orange bars).

Based on their properties as pseudo-quinone compounds, we studied the possible metabolization of indolone derivatives by hQR2 by analyzing the production of radicals with EPR and 5,5′-dimethyl-1-pyrroline-*N*-oxide (DMPO) spin traps, as previously reported [20]. Menadione, a natural non-specific substrate of quinone reductases, and *N*-[2-(2-methoxy-6*H*-dipyrido[2,3-a:3,2-e]pyrrolizin-11-yl)ethyl]-2-furamide (S29434), a specific inhibitor of hQR2 [23], served as controls for these experiments (structures in Figure 1). As illustrated in Figure 3A, the generation of radicals was highly compound-dependent as shown for compounds **1** and **11**. In both cases experimental spectra (Figure 3A, top) are compared with simulated spectra (Figure 3A, bottom). Some spectra fit the pattern for a single type of radical (Figure 3A, left, compound **11**), and others required the superposition of signals that corresponded to two or three types of radicals (Figure 3A, right, compound **1**). The EPR signals first depend on the stability of the reduced form, and then, on the kinetics of its re-oxidation. In these cases, as demonstrated with compound **11**, the main radicals detected were hydroxyl radicals, which produced four peaks characteristic of the spectra for the [DMPO-OH]^•^ adduct. In some cases (e.g., compound **1**), we also detected superoxide radicals (e.g., [DMPO-OOH]^•^ adduct) and methyl radicals (e.g., [DMPO-CH_3_]^•^ adduct) that resulted from a secondary reaction with the hydroxyl radical and dimethylsulfoxide (DMSO), which was used to dissolve the compounds [20,21].

Figure 3B compares the antiplasmodial activity of each compound to the quantity of radicals produced, evaluated as the sum of the double integrations of each EPR peak. Very interesting and at the heart of this work, the series that were inactive against *P. falciparum* [2-aryl-3*H*-indol-3-ol (compound **13**), *N*-hydroxyindoles (compounds **14** & **15**)] and 1-*H*-indoles (compounds **16**, **17**, **18**) do not possess a pseudo-quinoid structure and so did not generate radicals during metabolization by hQR2. Considering the active compounds, the trend was an increase in the production of radicals when the IC_50_ decreases. These results provided a link between the radicals produced upon hQR2 activity (i.e., the reduction of compounds by the quinone reductase) and their antiplasmodial activity.

### 2.3. Free Radicals Produced by Cellular Metabolization of Indolone Derivatives

Given the results obtained for the pure hQR2 protein, we conducted similar experiments in healthy RBCs with fewer active compounds. Here, we examined whether radicals could be produced upon activation of the naturally occurring hQR2 present in RBCs.

**Figure 4:**
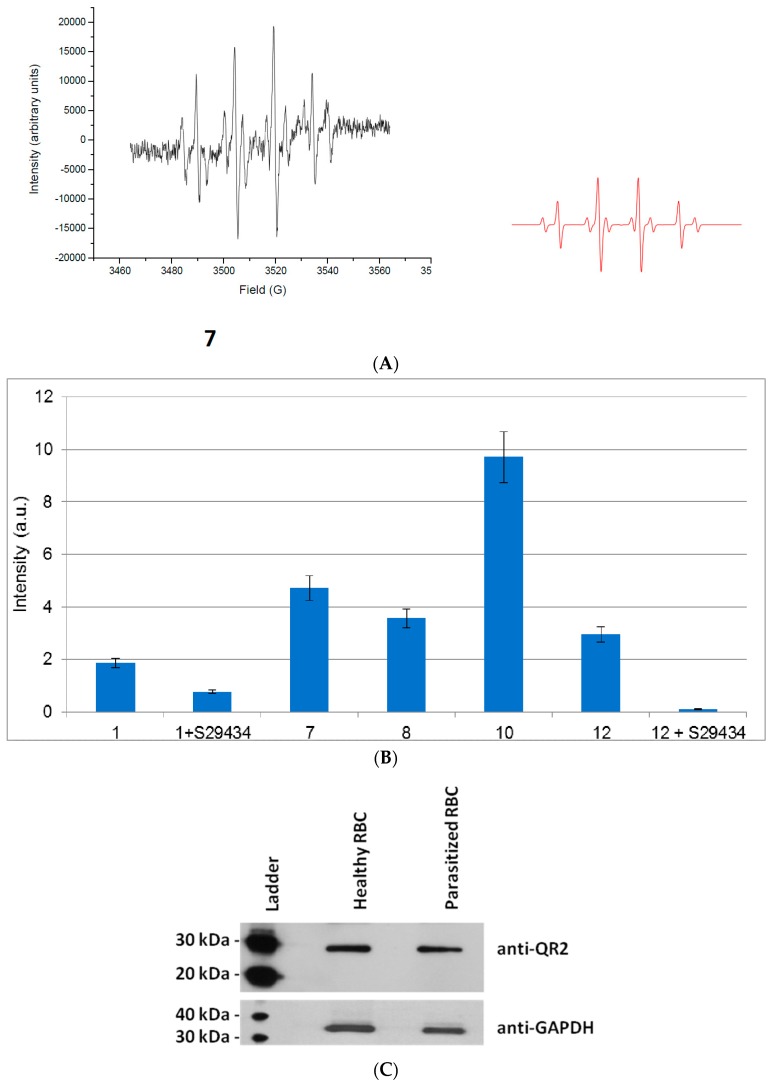
Free radicals produced by metabolization of indolone derivatives in red blood cells. (**A**) EPR spectra recorded (left) after adding **7** to red blood cells, compared to the simulated spectrum (right, in red); (**B**) Comparison of reactive oxygen species (ROS) production for compounds selected from the two sets of active series. Cells (5 × 10^6^) in DPBS buffer were treated with an indolone derivative substrate (100 µM), *N*-benzyldihydronicotinamide BNAH (100 µM), and DMPO (50 mM) (6% DMSO in the final mix). ROS production was evaluated by double integration of the area under the peaks. In two cases, cells were pre-incubated with S29434 (20 µM) for 30 min before adding compounds **1** and **12**; (**C**) Comparison of the expression of hQR2 expression in healthy versus parasitized red blood cells by western blot.

**Figure 5:**
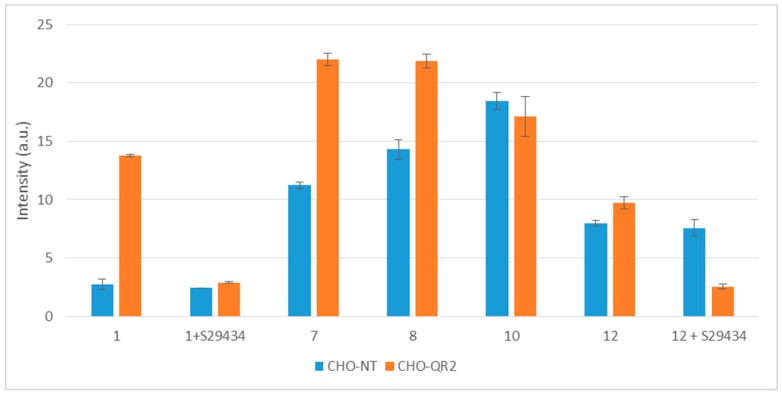
Free radicals produced by metabolization of indolone derivatives in CHO cells. Radical production was measured after two different CHO cell lines were treated (2 min incubation) with indolone derivatives selected from the two active series. One cell line overexpressed hQR2 (CHO-QR2, orange bars) and the other was not transfected (CHO-NT, blue bars), and therefore, expressed only endogenous QR2 levels. Cells (5 × 10^6^) were treated with the indicated indolone substrate (100 µM), *N*-benzyldihydronicotinamide (BNAH) (100 µM), and DMPO (50 mM). Radical production was evaluated as the double integration of the area under the EPR peaks. When indicated, cells were pre-incubated for 30 min with S29434 (20 µM).

The metabolization of these active compounds by RBCs gave rise to EPR spectra with various intensities (Figure 4A). We observed four main peaks characteristic of the [DMPO-OH]^•^ adduct, which originated from the decomposition of the [DMPO-OOH]^•^ formed by trapping superoxide radicals. We also detected smaller amounts of hydroxyl radicals that were converted into methyl radicals by reacting with DMSO (Figure 4A). The amounts of radicals produced by RBCs treated with different compounds are illustrated in Figure 4B. Addition of 20 µM of the hQR2 inhibitor, S29434 before addition of an antimalarial compound caused a reduction of 30% to 50% in the EPR signal intensity. This demonstrated the significant role played by the hQR2 protein in RBCs in reducing the pseudo-quinones, and the subsequent radicals produced when the compound was re-oxidized. It should be noted that other flavoenzymes, as glutathione reductase could reduce the INOD derivatives [26,27,28]. To confirm that hQR2 could be involved in the antimalarial properties of these compounds, we performed western blot analyses to measure hQR2 expression in healthy and parasitized RBCs (Figure 4C). The results showed that *P. falciparum* did not affect the hQR2 expression pattern, because hQR2 was expressed in both control and infected RBCs. The same experiments were then performed in Chinese hamster ovary (CHO cells), which permitted modulation of hQR2 expression. We monitored the generation of oxygen radicals in the extracellular medium of CHO cells that overexpressed hQR2 (CHO-QR2) and compared the findings with results obtained with CHO cells that were not transfected with the hQR2 construct (CHO-NT). Similar to our findings in RBCs, the metabolized compounds gave rise to EPR spectra generated by the trapping of superoxide radicals (not shown). The amounts of radicals produced from cells treated with the different compounds are shown in Figure 5.

The generation of radicals clearly varied, depending on QR2 expression and on the nature of the substrate tested and its IC_50_ value on *P. falciparum*. The addition of indolone-*N*-oxide antimalarial compounds led to more intense EPR signals in the CHO-QR2 cell line than in the CHO-NT cell line. In contrast, adding the inhibitor, S29434, significantly reduced the signal intensity (80% for **1**), which demonstrated a greater ability for compound **1** to be re-oxidized, compared to the other molecules tested. The highest EPR signal intensities were observed for the most active compounds (compounds **7** and **8**) which had smaller IC_50_ values. Compounds **10** and **12** of the IND series produced similar amounts of radicals in the two cell lines. When cells were pre-incubated with S29434, the signal intensity obtained with compound **12** was strongly reduced in CHO-QR2 cells, while it had no effect in the CHO-NT line. These results clearly confirmed the role of hQR2 in the reduction of the compounds tested and in the production of radicals, which are responsible for their antimalarial properties.

### 2.4. Intracellular Metabolization of the Compounds

We studied the role of hQR2 in the intracellular metabolization of indolone derivatives by following re-oxidation of the pseudo-hydroquinone (QH_2_ into Q) generated after enzymatic reduction by the impermeable membrane redox indicator, ferricyanide. Metabolization was measured as a decrease in the absorbance of potassium ferricyanide Fe^3+^, when it was reduced into Fe^2+^, upon interacting with the reduced substrate (pseudo-hydroquinones) [21]. Ferricyanide acts as a sink for pseudo-hydroquinones; it minimizes their intracellular re-oxidation via mitochondrial electron transport complex III and their conjugation via other phase II enzymes [29]. The decrease in absorbance of ferricyanide can be followed by UV-visible spectroscopy, and it indirectly reflects the rate of reduction of the tested substrates.

**Figure 6:**
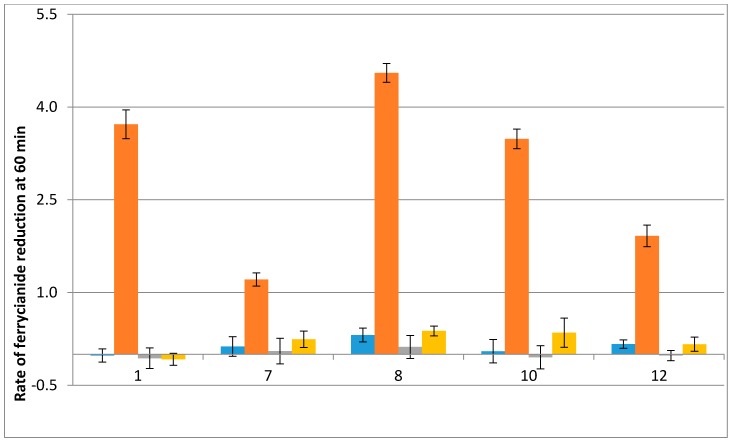
Rates of ferricyanide reduction reflect the metabolization of indolone derivatives in CHO cells. Ferricyanide reduction rates were measured (mean % reduction ± SD) in two cell lines treated for 60 min with potassium ferricyanide (600 µM), BNAH (100 µM), and the indicated indolone substrates (100 µM). One cell line overexpressed hQR2 (CHO-QR2, orange bars) and the other was not transfected (CHO-NT, blue bars), and therefore, expressed only endogenous QR2 levels. The % reduction was evaluated by comparing absorbance in treated cells to the absorbance measured in the absence of substrates. Cells were pre-incubated for 24 h with the QR2 inhibitor S29434 (20 µM): CHO-QR2 + S29434, yellow bars; CHO-NT + S29434, grey bars.

Given that RBCs are rich in iron complexes, this methodology was unsuccessful in RBCs. However, we obtained clear results with CHO-cells (Figure 6). The CHO-NT cells showed little or no decrease in absorbance, regardless of the substrate used. However, when CHO-QR2 cells were treated with pseudoquinones, a strong decrease in ferricyanide absorbance was observed. The INODs, compounds **1** and **8**, caused the greatest Fe^3+^ reductions, 37% and 45% in CHO-QR2 cells, respectively (Figure 6). Compound **10** also caused a 35% reduction in potassium ferricyanide.

The other compounds tested caused less than 20% reductions. When CHO-NT cells were pre-incubated for 24 h with S29434, little or no decrease in the rate of Fe^3+^ reduction was observed; this result indicate that this cell line expressed low levels of endogenous QR2. In contrast, in CHO-QR2 cells, pre-incubation with S29434 almost totally inhibited the metabolization of all compounds tested. Because S29434 is a very potent QR2-specific inhibitor, its effect on the reduction of substrates in cells that overexpressed QR2 demonstrated the role that QR2 played in the intracellular process involved in metabolizing the various substrates tested. Of note, the 24-h pre-incubation with S29434 did not affect cell viability.

These observations showed that hQR2 played an important role in the bioreduction of antimalarial compounds. INODs and INDs appeared to be activated by hQR2, producing active metabolites such as superoxide and other radical species. These results are supported by the observation that reductions in Fe^3+^/Fe^2+^ were proportional to the amounts of two-electron-reduced substrates present in the extracellular medium.

## 3. Materials and Methods

### 3.1. Chemicals

All the indole derivatives (structures shown in Figure 1) were prepared in our laboratory, as previously described: 2-aryl-3*H*-indol-3-one-*N-*oxides (INODs) [4], 2-aryl-3H-indol-3-ones (INDs) [30], 2-aryl-3*H*-indol-3-ol and *N*-hydroxyindoles and 1-*H*-indoles [7]. The *N*-[2-(2-methoxy-6*H*-dipyrido[2,3-a:3,2-e]pyrrolizin-11-yl)ethyl]-2-furamide (S29434) was prepared as described by Mailliet et al. [31].

Other chemicals included: Dulbecco’s Modified Eagle Medium (DMEM), fetal bovine serum (FBS), Dulbecco’s phosphate buffered saline (DPBS), phosphate buffered saline (PBS), potassium ferricyanide, superoxide dismutase, tris(hydroxymethyl)aminomethane buffer solutions (Tris buffer), *n*-octyl-β-glucopyranoside (octyl-GP), menadione, dicoumarol, and flavine adenine dinucleotide (FAD), all from Sigma-Aldrich-Fluka Co. (Saint Quentin Fallavier, France); 5,5′-dimethyl-1-pyrroline *N*-oxide (DMPO) from Dojindo (Kumamoto, Japan); 5(6)-carboxy-2′,7′-dichlorodihydrofluorescein diacetate from Invitrogen (Saint Aubin, France); *N*-benzyldihydro-nicotinamide (BNAH) from TCI Europe (Zwijndrecht, Belgium); and nicotinamide riboside (NRH) from Servier’s Research Institute (IdRS; Croissy-sur-Seine, France). Solvents were obtained from Thermo Fisher Scientific (Illkirch, France). We used a Milli Q^®^ water purification System (Millipore, St. Quentin, France) to obtain high-purity distilled water, which was necessary in preparing all aqueous solutions. Compounds were dissolved with sonication in an ultrasonic bath (Elma^®^, Friedrichshafen, Germany).

The enzyme 6his-hQR2 was produced by transfecting Sf9 cells with the PCR-amplified pcDNA3.1(+)/*hs*QR2 plasmid, as described by Nosjean et al. [32]. The vector was provided by IdRS.

The expression of hQR2 in RBCs was performed by Vectalys. Human blood was obtained from EFS (Toulouse, France). Naive Chinese hamster ovary (CHO-k1-NT) cell lines that overexpressed hQR2 (CHO-k1-QR2) were purchased from Vectalys (Ramonville Saint-Agne, France).

### 3.2. hsQR2 Enzymatic Activity

The affinity of compounds **1**, **5**, **8**, and **10** for hQR2 was measured with kinetics assays, performed as previously described by Mailliet et al. [31]. Briefly, the reaction was performed in 50 mM Tris-HCl, containing 1 mM *n*-octyl-β-glucopyranoside and 500 nM FAD. 20 mM stock solutions of substrates and BNAH in 100% DMSO were prepared and both diluted to 2 mM in DMSO or in buffer respectively. In a 96-well black plate, BNAH (100 µM) was mixed with *hs*QR2 (0.5 nM), and each antimalarial compound in a total volume of 200 µL with a constant concentration of 5.5% DMSO. The compounds added were diluted in pure DMSO to investigate the range of 100 µM to 0.39 µM (100; 50; 25; 12.5; 6.25; 3.125; 1.5625; 0.78125; 0.390625 µM). The hQR2 enzymatic activity was detected as a decrease in BNAH fluorescence, measured at 440 nm, with an excitation at 340 nm, which corresponded to its oxidation within the enzyme. This reaction was followed at 37 °C with a Safas Xenius^®^ plate reader (Monaco). In parallel, control reactions were performed with only BNAH and either the substrate or the hQR2. All experiments were performed in independent triplicates. The slope of the decrease in fluorescence was determined mathematically and expressed in nmol/min/nmol of protein corresponding to the maximal velocity of the catalytic oxido-reduction reaction. From this slope, *K*_M_, *V*_max_, and their corresponding standard errors were determined through a nonlinear regression fit with PRISM software (GraphPad Software Inc., San Diego, CA, USA).

### 3.3. Co-Crystallization of hQR2 with Bound Substrate—The RX Structure

We prepared 160 µM purified hQR2 in 20 mM Tris-HCl pH 8, 150 mM NaCl, 10% glycerol, and FAD was added at a final concentration of 240 µM. This FAD-enriched QR2 was subsequently mixed with compound **8′** or compound 10, at a final concentration of 1.5 mM in the presence of 8% DMSO. This mixture was incubated for 1 h on ice. The supernatant was recovered, the buffer was exchanged, and the protein was concentrated with a 10 kDa-PES Centricon^®^ (Merck Millipore Corp., Darmstadt, Germany) to a final concentration of 1.6 mM. Samples were centrifuged at 13,000× *g* for 15 min before crystallization. Hanging drops of hQR2-FAD were formed in the presence of either compound **8′** or compound 10 by mixing equimolar ratios of the complex and the reservoir, which contained either 1.6 M ammonium sulfate, 100 mM bicine pH 8 & 1.4 M ammonium sulfate or 100 mM bicine pH 7.5. The drops were immediately seeded with the micro-seeding technique. The seed stock was obtained from a 1.6-μL crystallization drop of the apo QR2-FAD complex screen, diluted with 18 µL of the crystallization solution composed of 1.4 M ammonium sulfate in 100 mM Hepes, pH 7. The first crystals appeared overnight at 20 °C. We selected the best crystals and cryo-protected them in the crystallization solution, supplemented with 20% ethylene glycol, and then flash-cooled them in liquid nitrogen. All diffraction data were collected on the beamline Proxima1, at the Synchrotron SOLEIL (Paris, France). The X-ray data for QR2-FAD bound to compound **10** or to compound **8′** were collected at a resolution of 1.9 Å and 1.5 Å, respectively. Diffraction intensities were indexed and scaled with the XDS program [33]. Both structures were solved by the molecular replacement method (PHASER from the CCP4 suite) [34,35], where the coordinates of the QR2-FAD complex structure were used as the search model (PDB ID: 1QR2). The constraints for the compounds **10** and **8′** were created with the Grade program (Global Phasing) [36].

Model building and refinement were carried out with COOT [37] and Buster [36]. Data collection and refinement statistics are summarized in Table 3. The atomic coordinates and the structural factors of QR2-FAD-10 or QR2-FAD-8′ have been deposited in the Protein Data Bank (http://www.rcsb.org), under the accession codes: 4XDH and 4XDG, respectively.

### 3.4. In Vitro Cultures of P. falciparum

Human erythrocytes obtained from EFS (group O±), were washed with RPMI medium to remove plasma and leukocytes. The chloroquine-resistant *P. falciparum* strain, FcB1, was maintained in vitro, with human erythrocytes in RPMI 1640 medium (BioWhittaker, Cambrex, Belgium), which contained l-glutamine, 25 mM GEPES, and 10% human serum (EFS), as previously described [4]. Human erythrocytes were maintained routinely at 2% parasitaemia in culture medium (hematocrit: 2%) in a controlled atmosphere (5% CO_2_, 100% relative humidity) and synchronized with 5% d-sorbitol lysis every 48 h [38].

### 3.5. Isolation of Parasitized Red Blood Cells (pRBCs)

The isolation and purification of pRBCs were performed as previously reported [39]. Briefly, we inserted MACS^®^ columns (25 LD columns, Miltenyi Biotec, Paris, France) into a MACS^®^ magnetic support (Quadro, Miltenyi Biotec, Paris, France) and filled the columns with RPMI. The blood from a parasite culture was deposited at the top of the column (1 mL at 50% hematocrit). Then, the LD columns were washed with RPMI until the eluent was apparently free of RBCs. The columns were removed from the magnetic support, and a volume of 2 × 2 mL of RPMI was added to the column to recover the eluent of parasitized erythrocytes (>80% parasitaemia). This eluent was then centrifuged (1600 rpm, 56 min) and the supernatant, removed. The pellet was lysed to extract the protein.

### 3.6. Cells Lysis and Protein Extraction

Pellets of 10 × 10^6^ erythrocytes and 10 × 10^6^ erythrocytes parasitized with *P. falciparum*, in the old, trophozoite stages, were washed with 500 µL cold DPBS (Sigma, St. Louis, MO, USA) and centrifuged (1500× *g*, 5 min, 4 °C). Supernatants were removed, and pellets were re-suspended in lysis buffer containing RIPA buffer (Sigma Aldrich), 1% protease inhibitors (Sigma Aldrich), and 1% phosphatase inhibitors (Sigma Aldrich). The resulting mixture was mixed on a vortex for a few seconds and incubated at 4 °C for 20 min. After incubation, the mixture was centrifuged (14,000× *g*, 15 min, 4 °C). The supernatant was recovered, and 30-µL aliquots were stored at −80 °C for western blot analyses.

### 3.7. Western Blotting

Total proteins (15 µg) were loaded onto an SDS-PAGE gel with a gradient of 4%–12%. The separated proteins were blotted onto a PVDF membrane. Western blots were probed with either an anti-QR2 (a.k.a. NQO2) antibody (H00004835-A01, diluted 1:500, Abnova, Taipei City, Taiwan) or an anti-GAPDH antibody (clone SIGMA-71.1, diluted 1:10,000, Sigma,) as a loading control. ECL signals were detected with the Pierce Fast Western Blot Kit (Pierce Biotechnology, Rockford, IL, USA).

### 3.8. Reactant and Substrate Preparations for the Free Radical Production Measurement

For acellular experiments, experiments were performed in Tris-β-octyl buffer (50 mM Tris-HCl and 1 mM octyl-GP) pH 8.5. We prepared a 1 M DMPO stock solution in deoxygenated water, which was stored at −80 °C until use. We prepared all substrates at 2.5 mM in DMSO. The BNAH was prepared at 20 mM in Tris-β-octyl buffer. For assays with the QR2 inhibitor, S29434, we prepared the compound at 12 mM in a stock solution in DMSO; for use in experiments, the stock solution was diluted 1:100 in Tris-β-octyl buffer.

For cellular experiments, all substrate solutions, co-substrates solutions (4 mM), and hQR2 inhibitor (S29434) solutions (1 mM) were prepared in DMSO. For experiments, 5 × 10^6^ cells/assay were cultured in DPBS and treated with final concentrations: DMPO 50 mM, substrates 100 µM, BNAH 100 µM. The inhibitor was incubated at 20 µM with cells for 30 min at 37 °C before taking a reading. We verified that adding 50 mM of DMPO to the cells did not trigger any production of free radicals.

### 3.9. EPR Spectroscopy Experiments on hQR2

All experiments were performed with hQR2 (20 µg/mL), DMPO (125 mM), the indolone substrates (125 µM), and BNAH (3 mM); for some assays, we added S29434 (5 µM). EPR spectra were acquired with a Bruker EMX-8/2.7 (9.86 GHz) equipped with a high-sensitivity cavity (4119/HS 0205) and a gaussmeter (Bruker, Wissembourg, France), at X-band and at room temperature. For analyses, we used a flat quartz cell, FZKI160-5 × 0.3 mm (Magnettech, Berlin, Germany). We performed data processing and spectrum simulation with WINEPR and SIMFONIA software (Bruker). Scanning parameters were: microwave power, 20 mW; sweep width, 100 G; sweep time, 83.88 s; time constant, 40.96 ms; magnetic field, 3460–3560 G; scan rate, 1.2 G/s; scan number, 5; modulation amplitude, 1 G; and modulation frequency, 100 kHz. We conducted the EPR spectra analysis as reported previously [20]. Each EPR spectrum comprises the intensity of the peaks on the *y*-axis in arbitrary units (a.u.), and the magnetic field on the *x*-axis in gauss (g). The scale of the magnetic field is not included for reasons of simplicity because it is not used in this study. The features used are the signal intensity measured by integrating the area under all peaks and the signal shape with the number of peaks.

### 3.10. Reactant and Substrate Preparations for the Intracellular Metabolization Assay

The indolone compounds **1**, **7**, **8**, **10** & **12** as well as the BNAH stock solutions were prepared in DMSO (at 4 mM), then diluted 8-fold in DPBS (0.5 mM). Potassium ferricyanide was prepared at 1.2 mM in DPBS. For assays with the S29434 inhibitor, 1 mM solutions were prepared in DMSO, and then, diluted in medium to obtain the final concentration of 20 µM. Cells were incubated with the inhibitor for 24 h before withdrawing the inhibitor and rinsing the cells in medium.

### 3.11. Cellular Activity Assays

All experiments were carried out with confluent and adherent CHO cells on 96-well plates. Cytoplasmic ferricyanide reduction was monitored with UV-visible spectroscopy by measuring the absorbance of ferricyanide for 1 h at 421 nm (ε = 1.0 mM^−1^·cm^−1^), then every 10 min with a plate reader (EON, Biotek, Colmar, France). Cells were maintained at 37 °C with gentle orbital agitation between readings. Substrate-mediated ferricyanide reduction was measured after treating the cells with final concentrations of 100 µM of substrate and co-substrate and 600 µM of ferricyanide. Background reduction rates were measured in cells treated with ferricyanide alone. The background rate of cell-mediated ferricyanide reduction was subtracted from the measurements carried out in the presence of each substrate. All experiments were conducted in triplicate, and all results are expressed as the mean ± standard deviation (SD).

## 4. Conclusions

In the present work, we demonstrated that the anti-malarial activity of indolone derivatives is linked to their ability to serve as hQR2 substrates knowing that QR1 is not expressed in the RBC. Other reductases could be implicated in the biotransformation of indolone in the RBC. However, we have previously demonstrated that the two electron-reductase hQR2 can reduce a wide range of compounds even hardly reducible which is the case of indolone derivatives. Once reduced, their intrinsic instability led them to re-oxidize, in the presence of dissolved oxygen. This return to the original form produced free radicals. The production of radicals is fatal to the stability of parasitized RBCs and hence to the parasites while it induces no hemolytic effect in the healthy RBCs [8,30]. This result is highly interesting, because, to date, it was assumed that the activity of antimalarial compounds arose from their inhibition of hQR2. Based on our results, hQR2 can be considered a target of great interest in the design of new antimalarial compounds that act as hQR2 substrates.

## Figures and Tables

**Figure 1:** Structures of the compounds tested in this study.

**Table:** 

	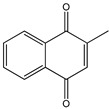 Menadione		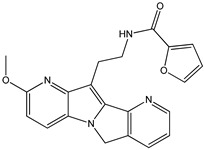 S29434	
Indolone-*N*-oxide (INODs)	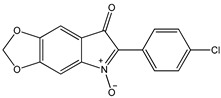 **1**	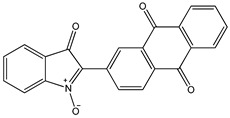 **2**		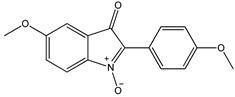 **3**
	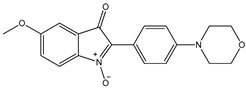 **4**	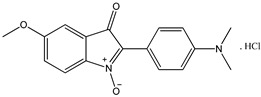 **5**		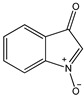 **6**
	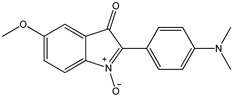 **7**	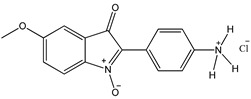 **8**		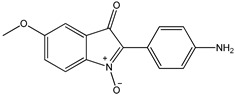 **8’**
2-Aryl-3*H*-indol-3-ones	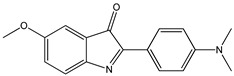 **9**	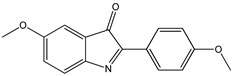 **10**		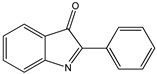 **11**
	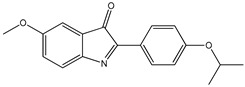 **12**			
2-aryl-3*H*-indol-3-ol	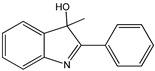 **13**			
*N-*hydroxyindoles			
	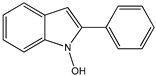 **14**	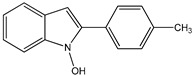 **15**		
1*H*-indoles	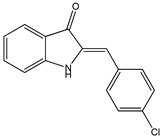 **16**	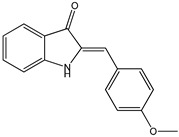 **17**		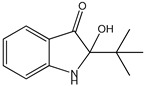 **18**

**Table 1:** Kinetic constants of hQR2 for the substrates tested. Results are the mean ± SD of three separate determinations.

Substrate ^a^	*V*_max_ (µmol·mL^−1^·min^−1^)	*K*_M_ (µM)
Menadione	4264 ± 134	10 ± 1
1	789 ± 39	47 ± 5
8	1471 ± 98	12 ± 2
10	730 ± 59	21 ± 4
12	1255 ± 93	30 ± 6

^a^ All substrate stock solutions (20 mM) were prepared in 100% DMSO and then diluted in Tris-β-octyl buffer (50 mM Tris-HCl, 1 mM octyl-GP) pH 8.5 (DMSO final 5.5%) and reactions are followed at 37 °C.

**Table 2:** Interactions established by compounds **8′** and **10** in the human quinone reductase 2 flavin adenine dinucleotide (FAD) binding pocket.

Nature of the Interaction	Monomer	Compound Atom	Protein Atom	H_2_0	Distance (Å)
	**hQR2-FAD Bound to Compound 10, PDB Entry: 4XDH**				
Hydrogen bond	A	O12/233	/	W199	2.73 Å
		O4/233	/	W142	2.84 Å
	B	O12/233	/	W180	2.72 Å
π–π stacking	Interaction between the isoalloxazine ring and compound **10**				
	**hQR2-FAD Bound to Compound 8’, PDB Entry: 4XDG**				
Hydrogen bond	A	O10/233	/	W459	3.33 Å
		O13/233	/	W175	2.76 Å
	B	O13/233	/	W558	2.66 Å
π–π stacking	Interaction between the isoalloxazine ring and compound **8′**				

**Table 3:** Data collection and refinement statistics.

Data Collection and Refinement Statistics		
	*h*QR2-FAD Bound to Compound 10, PDB Entry: 4XDH	*h*QR2-FAD Bound to Compound 8′, PDB Entry: 4XDG
X-ray source	SOLEIL synchrotron, Proxima1	SOLEIL synchrotron, Proxima1
Oscillation range (°)	0.1°	0.1°
Number of frames	2000	2000
Exposure (s)	0.1	0.1
Detector distance (mm)	296.299	270.864
Wavelength (Å)	0.97857	0.97857
Space group	*P*2_1_2_1_2_1_	*P*2_1_2_1_2_1_
Unit cell parameters *a*, *b*, *c* (Å)	56.514, 84.030, 106.420, 90.000, 90.000, 90.000	56.487, 83.634, 106.375, 90.000, 90.000, 90.000
Resolution (Å)	50.0–1.9 (2.01–1.90)	50.0–1.5 (1.59–1.50)
*R*sym	20.2 (109.1)	19 (61.9)
*I*/s*I*	6.83 (1.61)	12.58 (3.11)
Completeness (%)	99.4 (97.6)	99.9 (99.5)
Multiplicity	7.32 (7.34)	7.31 (7.14)
Number of reflections	40641	81302
(XX) statistics for high resolution range		
**Refinement Statistics**		
Resolution (Å)	27.82–1.90 (1.95–1.90)	46.81–1.50 (1.54–1.50)
*R*work/*R*free (%)	17.09/19.88 (22.41/23.13)	16.63/18.66 (23.98/28.35)
Number of atoms (protein/waters)	3635/255	3727/524
Mean B-factor (Å^2^)	32.17	26.25
R.m.s deviations: Bond lengths (Å)/Bond angles (°)	0.010/0.99	0.010/1.02
